# Nothing wrong with me

**DOI:** 10.7554/eLife.93330

**Published:** 2023-10-12

**Authors:** Simone Brixius-Anderko

**Affiliations:** 1 University of Pittsburgh School of Pharmacy, Department of Pharmaceutical Sciences, Center for Pharmacogenetics Pittsburgh United States

**Keywords:** sparks of change, being neurodivergent in academia, equity diversity inclusion, neurodiversity, autism, research culture

## Abstract

An assistant professor and group leader explains how being diagnosed with autism in her early 40s changed her approach to being a scientist.

My youngest daughter has always been special, with a fantastic way of thinking and acting that society told us was “outside of the norm”. When she started to struggle at school, her therapist suggested that she may be autistic. As I read up on the topic, I started to wonder if perhaps I was as well; I received my autism diagnosis one year after she got hers. I was 41 years old at the time and a postdoc at the University of Michigan.

At first, I didn’t quite know what to do with this information. Growing up poor in 1980s Germany, I had always been the ‘socially awkward’ kid nobody really understood but many took advantage of. I had trouble connecting with my peers, often being told that I was too blunt. As an adult, I had carried the feeling that something was seriously wrong with me, which made other people simply not like me.

It was the therapist who assessed me who initially brought up how my autism may have contributed to the career I had built despite my difficult upbringing. I had dropped out of high school when I was 17 so I could work and leave my childhood home as soon as possible. I was 27 and a single mom of two when I started my master’s degree and embarked on my scientific career. I was surprised by what the therapist had said at first, but I came to see how this may have been the case. Being autistic had helped me rationalize hardship and remain focused on my goals, even when people had advised me to give up, or when I was going against what society thought someone like me should do. These conversations encouraged me to look at my path, and ultimately at myself, with a different perspective.

I thought back to how I had fallen deeply, madly in love with biochemistry when attending night classes to complete my high school degree. Nothing was more soothing to my mind than learning about glycolysis and all these beautiful, logical chemical reactions. As a child I had been obsessed with things like nuclear power or playing the flute, teaching myself everything I could about these topics to the point of forgetting the world around me. This ability to hyperfocus, which I now understand is one of my autistic traits, has become a great asset as a scientist. Sometimes, I simply don’t care about anything except what I’m working on. I can get a lot done in a short amount of time, though I can also pay the price by crashing afterwards.

Piece by piece, other things started to make sense in the light of my diagnosis, like my sadness and sheer disgust when I first realized that science often relies on the ability to network and sell yourself. Sure, I can occasionally be chatty, funny and entertaining; I can even make small talk if I’m in a really good mood! However, I have to be aware of the toll that this may take on me. In my autistic mind, sentences must be perfect before they are spoken, which can make it difficult to engage in a conversation. It doesn’t help that English is not my native language, so I’m often overly apologetic and quite self-conscious about how I talk. After a chat, I tend to frantically go over the interaction to evaluate whether I’ve behaved appropriately, worried that I may have missed a social clue and done something that could have been perceived as rude.

In retrospect, it’s no wonder why I’d been finding conferences so challenging. My sensory sensitivities are a gift when it comes to fully immersing myself into music, but they work against me when lights are too bright, rooms are too crowded, noises are too loud. Like many autistic people, I can also struggle to process auditory information. I can hear the words, but I just cannot make sense of them. This makes it difficult for me to follow talks, especially since captions are rarely available. When you add the pressure to network and socialize, it can all become overwhelming. Once, as a postdoc, I attended a two day conference where I didn’t have any time to myself to recharge my social energy. I became so exhausted that I had a meltdown, snapping at everyone and ultimately just running out of the venue. I was sobbing my eyes out on the bus home when I realized that a professor I knew well was sitting not far from me. When he asked if everything was alright, I was still so shut down that I could not answer. As expected, he had the kindest response when I emailed him later to apologize, but this interaction showed me that I needed to take care of myself better.

Ultimately, my diagnosis allowed me to realize that there is nothing wrong with me; my brain is just different. Most importantly, it helped me to be kinder to myself. I’m not rude for speaking my mind or missing social cues, and I’m not dumb for struggling to follow a conversation. I’ve learned to embrace my phoenix cycles of hyperfocusing and crashing out, which means sometimes taking mental health days to prevent autistic burnout. I know I need rest to recover from social interactions and that I deserve to take breaks: I enjoy conferences so much more now that I allow myself to go watch Shark Tank in my hotel room when I’ve reached my limit! I am teaching myself that I don’t have to smile only because I feel I should; that I shouldn’t hold back and hide who I am anymore.

This is still a work in progress. From an early age, girls are trained to make eye contact, to be quiet, soft and polite. I know for sure I wasn’t the only one brought to tears by America Ferrara’s famous monologue in the movie Barbie: “You have to never get old, never be rude, never show off, never be selfish, never fall down, never fail, never show fear, never get out of line.” Women on the spectrum often make a huge effort to mask their feelings so they can adhere to social norms and please others. We often don’t have much choice. We are literally told that the way we are doesn’t fit into society; more so in academia; more so in leadership positions. It’s no wonder why our suicide rate is so much higher, but also why we tend to not get diagnosed to start with. How often have I heard: “Your daughter smiles so much and is so polite. Are you sure she is autistic?” Even the way autism is assessed is still tailored for how it presents in boys. When I learned this, I was furious. How can the official testing process still be that sexist and outdated?

When I was a child, autism was only known from movies like Rain Man, and autistic people were depicted as mentally impaired. Autism awareness has improved since then, but gaps remain. Many people still believe that we aren’t empathetic for example, when the absolute opposite is true. I’ve been on the receiving end of many doubtful “Ahs” and “Ohs” when I tell people about my autism; apparently, I’m not ‘autistic enough’ because I can be social and outgoing. It hurts to see people making excuses to not provide accommodations for autistic students and staff because they do not “act autistic” — whatever that may mean.

I’m a passionate advocate for my daughter so I can address ignorance at her school, but I also believe that neurodiversity has to be normalized in academia. Since receiving my diagnosis, I have become aggressively open about being autistic and now I celebrate being different. It fills my heart when students and colleagues come to me to share their own stories of being neurodivergent. Around them, I feel seen and understood. Autistic researchers are smart, capable, and passionate mentors and teachers. There is so much we can bring to the table.

## About this article

This Sparks of Change article is part of a series of articles on being neurodivergent in academia, which includes a list of tips, resources and tools collated by neurodivergent scientists.

